# Lived experiences of parents of premature babies in the intensive care unit in a private hospital in Johannesburg, South Africa

**DOI:** 10.4102/curationis.v40i1.1698

**Published:** 2017-02-28

**Authors:** Erika Steyn, Marie Poggenpoel, Chris Myburgh

**Affiliations:** 1 Department of Nursing Science, University of Johannesburg, South Africa; 2 Department of Educational Psychology, University of Johannesburg, South Africa

## Abstract

**Background:**

Many of the 15 million premature babies born worldwide every year survive because of advanced medical interventions. Their parents have intense experiences when their babies are in the intensive care unit (ICU), and these have an impact on their thoughts, feelings and relationships, including their relationships with their premature babies.

**Objectives:**

The aim of the study was to explore and describe the lived experiences of parents of premature babies in an ICU.

**Method:**

Research design was qualitative, exploratory, descriptive and contextual. A purposive sample of parents with premature babies in an ICU in a private hospital in Johannesburg Gauteng in South Africa was used. Eight parents, four mothers and four fathers, married and either Afrikaans or English-speaking, were included in the study. Data were collected by conducting in-depth phenomenological interviews with them and making use of field notes. Trustworthiness was ensured by implementing the strategies of credibility, transferability, dependability and confirmability. Ethical principles such as autonomy, beneficence, non-maleficence and justice were adhered to throughout the research process.

**Results:**

Thematic analyses were utilised to analyse the data. Two themes in the experiences of parents with premature babies in ICU became apparent. Parents experienced thoughts, emotions and hope while their premature babies were in the ICU as well as challenges in their relationships and these challenges influenced their experiences.

**Recommendations:**

Mindfulness of intensive care nurses should be facilitated so that intensive care nurses can promote the mental health of parents with premature babies in the ICU.

**Conclusion:**

Parents with premature babies in the ICU have thoughts and emotional experiences which include hope and they affect parents’ relationships.

## Introduction

Approximately 15 million premature babies are born worldwide every year; 60% of them are born in Africa and South–Asia (Blencove et al. 2012:2162). Several maternal health factors play a role, including maternal diabetes and hypertension, increased maternal age and obstetric practices such as caesarean sections before term (World Health Organisation 2013). Another factor is the prevalence of acquired immune deficiency syndrome in a country. In South Africa the human immunodeficiency virus is found in one of every four people; as a consequence more premature babies are born in this group of mothers than others (World Health Organisation 2013).

Parental fertility problems are also on the increase worldwide. The treatment for fertility problems leads to a higher incidence of multiple babies and the premature birth of those babies (Chordas 2007:2). Not only are more babies born prematurely, but more babies with an extremely low birth mass are surviving (Fanaro & Vigi 2007:204–209).

When a premature baby is born physical, psychological and social preparation for a baby is interrupted and the mother of the premature baby might feel as if she is missing out on something (Payot et al. 2007:1492, Coppola & Cassibba, 2010). Premature babies often require substantial support because their organs are immature. This can affect the bonding process between mother and child (Siegel & Hartzell 2004:31, 102). All of these factors can lead to an emotional roller-coaster ride (De Rouck & Leys 2009:159, Fegran, Helseth & Fagermoen, 2008) where parents experience powerful emotions. It is at this time that parents have to have hope for the survival of their premature babies. But they may at the same time realise that their babies can die.

In an intensive care unit (ICU) situation parents play a lesser role in the care of their babies. Not only are other people such as neonatal nurses and neonatologists more involved in looking after their babies than themselves, but their babies’ condition may fluctuate which will make it difficult for the parents to know what the future will bring. This might cause the experience of parents with premature babies in the ICU to be traumatic (Holditch-Davis et al. 2003:161–171; Lefkowitz, Baxt & Evans 2010:230–237; Vanderbilt et al. 2009:50–56).

These traumatic experiences can have a long-term effect on the mental health and functioning of the parents of premature babies (Siegel & Hartzell 2004:31, 102). The parents’ experiences of their premature babies in the ICU can have an impact on the normal attachment process (Fegran, Fagermoen & Helseth 2008:810–816). Adequate bonding is the foundation for healthy parent–child relationships (Siegel & Hartzell 2004:125). It is also the basis for the premature babies’ mental health. It is therefore important for parents and their premature babies, as well as their families and the community at large, to use parents’ experiences of their premature babies in an ICU to facilitate their mental health.

### Problem statement

Prematurely born babies require special care in ICUs. They are taken care of by professional people. Their parents have different experiences of their babies being in the ICU. The researcher has heard from parents she had worked with in her private practice that they are generally of opinion that while their premature babies were adequately cared for in ICUs, parents were expected to deal with their own strong emotions on their own. This was often unintentional but related to the workload of intensive care staff. This is also supported by literature in this regard (De Rouck & Leys 2009:160; Lou, Pedersen & Hedegaard 2009:567–573). These experiences can have a lasting effect on the mental health of the premature babies, their parents and ultimately the society that they live in. The research question therefore is: ‘What is the experience for parents of premature babies in intensive care units?’

### Aim and objective of the research

The aim of this research was to gain understanding of parents’ experiences of having premature babies in an ICU.

The objective of this study was to explore and describe the experiences of parents of premature babies in an ICU.

## Research design and method

### Research design

A constructivist philosophy of science was adhered to. This is based on observation and scientific study. It says that people construct their own understanding of the world through what they experience and their reflection thereof (Concept to Classroom 2004). A qualitative research design (Burns & Grove 2011:20; Shank 2006:40) was chosen to explore and describe what the experiences are of parents of premature babies in the ICU.

### Research method

An interpretative phenomenological approach was taken to understand and describe the parents’ lived experiences to have premature babies in an ICU (Callary, Rothwell & Young 2015:63; Larkin, Watts & Clifton 2006:113). This design promotes the researcher’s understanding of the meaning that they attach to their experiences however similar or different they may be.

### Population and sample

The population of this research study consisted of eight parents, four married couples, four mothers and four fathers with premature babies in an ICU in a private hospital in Johannesburg, Gauteng, South Africa.

Purposive sampling (Chinn & Kramer 2011:224; Shank 2006:30) was used to select parents who were information-rich on what their experiences were of having premature babies in the ICU. Sampling criteria were that they were married parents of premature babies in an ICU and Afrikaans or English-speaking. The researcher with the assistance of the professional nurses in the ICU selected the parents whose premature babies were in the ICU from 4 to 6 weeks and would still be in the ICU for another 8 weeks.

### Data collection

Eligible parents, selected with the assistance of the professional nurses in the ICU, were contacted and invited to take part in the research study. The researcher explained to the parents telephonically what the research entailed. Appointments were scheduled for individual in-depth phenomenological interviews with the parents who decided to participate in the research. These in-depth interviews were conducted at the following places: in the ICU (by request of the mother); at the homes of two of the couples, one of the mothers and one of the fathers; and at the consulting room of the researcher (by request of the father). During the in-depth interviews the researcher built a trust relationship with the participants by showing respect, listening carefully and clarifying any issue that was not clear. Interviews were conducted until data saturation (Keele 2011:49) was reached. The question that was posed to the parents was: ‘How is it to have your premature baby cared for in the intensive care unit?’ Data saturation is reached when no new information or new themes emerge from the analysis of the data (Guest, Bunce & Johnson 2006:59–82; Keele 2011:49). Interviews were audio-taped after permission was obtained from the participants. Field notes (Polit & Beck 2014:354) were made of observations made by the researcher after the in-depth interviews were conducted.

### Data analysis

The interviews were audio-taped with the written permission of the parents. Thereafter they were transcribed and analysed, using Tesch’s thematic coding (Creswell 2007:154–155, 2013:243–244). Using this method the researcher carefully read the transcribed interviews and field notes to get a sense of the experiences of parents with premature babies in the ICU. Words relevant to parents’ experiences were identified and coded. After they had been described they were translated into themes (Patton 2014:453). The themes were then supported by the parents’ direct quotations. Direct quotations in Afrikaans were translated into English at that point. An independent coder was used during the data analysis stage of the research study. A consensus discussion between the researcher and the independent coder was conducted after the data analysis was conducted. A literature control was performed to contextualise the results.

### Ethical considerations

Ethical clearance for this research was obtained from the Faculty of Health Sciences Research Ethics Committee of a university, ethics clearance number AEC 74/2009. The researcher also got permission to conduct the research study from the private hospital and the group it belongs to. Ethical measures like respect for autonomy, non-maleficence, beneficence and justice were adhered to throughout this research (Dhai & McQuoid-Mason 2011:43–44). Autonomy was adhered to by discussing the research with the unit manager at the ICU, who then discussed the research study with the parents who decided to take part in the research. The parents were also assured by the researcher that they could withdraw at any stage without any consequences to them. All the parents participated voluntarily to the research study. Psychological support was available should the parents have needed it after the interviews were conducted. The interviews were anonymous and confidential. Parents gave permission for them to be audio-taped. Data obtained were kept locked up in a cupboard in the researcher’s room and only the researcher had access to it. Data will be destroyed 2 years after publication of this research.

### Trustworthiness

Truth value is ensured by establishing credibility (Krefting 1991:215; Patton 2014:106). The researcher made use of prolonged engagement, field notes and triangulation of data (Creswell 2007:208) obtained by way of the in-depth interviews and field notes made of observations made during interviews. Findings were also discussed with peers and colleagues experienced in qualitative research techniques. Reflexivity is part of the qualitative research approach (Denzin & Lincoln 2011:124; Krefting 1991:219). The researcher reflected this in the auto-ethnographic interview conducted by her study leaders and the field notes made.

Field notes reflected feelings, thoughts, frustrations and problems experienced during the research process (Krefting 1991:218; Shaw 2010:240). Transferability was achieved by a dense description of the demographics of the participants and the results of the in-depth interviews supported by direct quotations from participants. Dependability was established with the dense description of the research design and process. An independent coder did a coding procedure on the data without her having any knowledge of the researcher’s interpretation of the results. Confirmability was achieved by providing a chain of evidence of the whole research process.

## Results

Two major themes were identified during the data analysis. They reflected the lived experience of parents with premature babies in the ICU. These themes and categories are given in Table 1.

**TABLE 1:** Themes and categories of parents’ experiences of having a premature baby in the intensive care unit.

Themes	Categories
1. Parents experienced thoughts, emotions and hope while their premature babies were in the ICU.	Parents experienced thoughts and emotions while their premature babies were in the ICU. Parents expressed and experienced hope for their babies in the ICU.
2. Parents experienced challenges in their relationships which influenced their experiences as parents or, alternatively, the parents’ experiences of their premature babies in the ICU might influence their relationships.	Parents had challenging experiences in relation to themselves. Parents had challenging experiences in relation to their babies. Parents had challenging relationships in relation to each other. Parents had challenging experiences in relation to the nursing staff. Parents had challenging experiences in relation to the medical staff. Parents had challenges with other parents who also had premature babies in the ICU. Parents had challenging experiences with family and friends. Parents’ spirituality was challenged by their experience to have a premature baby in the ICU.

ICU, intensive care unit.

Both themes and their categories will now be discussed. Relevant quotations from the interviews with parents will be used as well. Key to participants’ abbreviations: MW: Married to DW, Afrikaans-speaking, female, 30–34 year age group, lower socio-economic class, second premature baby and housewife. DW: Married to MW, Afrikaans-speaking, male, 30–34 year age group, and lower socio-economic class, second premature baby, employed by a large company. LvS: Married to CvS, Afrikaans-speaking, female, 35–40 year age group, upper middle socio-economic class, housewife and parent of premature triplets of which two babies survived. CvS: Married to LvS, Afrikaans-speaking, male, 35–40 year age group, upper middle socio-economic class, and parent of premature triplets of whom two babies survived, contracted his services to different organisations. CB: Married to MB, Afrikaans-speaking, female, 35–40 year age group, middle socio-economic class, on maternity leave at the time of the interview, mother of micro-premature baby. MB: Married to CB, Afrikaans-speaking, male, 35–40 year age group, middle socio-economic class, self-employed, father of micro-premature baby. CE: Married to GE, English-speaking, female, 30–35 year age group, middle socio-economic class, on maternity leave at the time of the interview. GE: Married to CE, English-speaking, male, 30–35 year age group, middle socio-economic class, self-employed.

*Theme one*: Parents experienced thoughts, emotions and hope while their premature babies were in the ICU.

Parents experienced and expressed strong thoughts and emotions while their premature babies were in the ICU. These thoughts and emotions influenced each other. They were ill-prepared for and traumatised by how vulnerable and fragile their babies surrounded by technology in the ICU, appeared to be. They reported feelings like distress, guilt, fear, frustration, envy, anger, jealousy and sadness. They experienced ambivalence in their feelings.

‘I did not know what to expect, when they said she weighed, what was it? I think it was 600 g or something. When I saw her it was different.’ (MB)

‘It was so bad to see him with all those things on him and he looked so helpless. It was so heavy and difficult for me.’ (MW)

‘Do you hold her the right way, can you do it? Can you do it? It was so difficult, she was so little.’ (CB)

‘It is nice to see the other babies going home because then you know yours will go too.’ (MW)

They found it difficult to express their emotions and accompanying thoughts. Parents experienced and expressed hope for their premature babies in the ICU. Parents became increasingly hopeful as they were able to take care of their premature babies and they reached some milestones. Getting timeous, accurate information and encouragement from medical and nursing staff fuelled their hope.

‘The nursing sisters who work with her, every day reassure you that she is normal.’ (CB)

‘I feel I made them ill.’ (LvS)

‘I mean to take as an example because she is wearing clothes now … one can become excited because of that.’ (CB)

Parents’ challenges in their various relationships also played an important role in their experiences of their premature babies in the ICU. These will be discussed in the second theme.

*Theme two*: Parents experienced challenges in their relationships; these challenges influenced their experiences as parents.

Parents had challenging experiences in relation to themselves, their premature babies, their spouses, nursing staff, medical staff, other parents of premature babies in the ICU as well as family and friends. Parents reported that their spirituality was also challenged by their experiences of having a premature baby in the ICU.

Every parent’s experiences were unique to them. They experienced losses like their previous identities, life-styles and routines. They also had to let go of the expectations that they may have had and adjust to a new reality. They had recurring thoughts and their self-esteem was challenged by their experiences.

Some parents expressed sadness that their babies were born prematurely and they were able to do so little for and with their babies. Parents expressed the unrealistic expectations that they had of themselves and their babies. They experienced being over-protective over their babies. Parents were not sure whether their babies would live, if they as parents were doing the right thing or if they as parents were competent parents.

‘The first alarm goes off and you get a terrible fright because you don’t know … Does your baby not breathe because of something that you did while you were holding her.’ (CB)

As spouses they had different expectations of each other and different experiences of how it was for the other parent to have a premature baby in the ICU, but they were often unable to express their needs to their spouses. They assumed different roles towards each other in terms of their premature babies. They relied on each other for support and showed their support in different ways.

‘I could bounce off emotions with him. I think I was quite fortunate to get the support that I had from my husband. The only person I could really share it with was my husband.’ (CE)

The better the relationship between the parents of the premature babies and the nurses in the ICU, the more the parents trusted the nurses and the more they felt secure about their babies’ treatment. Nurses in the ICU had opportunities to educate parents about parenting skills, their premature babies and their treatment; parents wanted nurses to share information with them on their premature babies’ progress, treatment and well-being and to explain medical terminology. Some parents trusted the medical and nursing staff and expressed their belief that their babies were well looked after, while other parents were concerned that some information was withheld from them.

‘You know the nursing sisters are trained to answer you but you are still sometimes unsure about what they are saying or how they explain things.’ (LvS)

Parents’ experiences of nurses’ attitudes and their interpersonal skills played an important role in the relationship the parents had with the nurses in the ICU. Nurses were seen as a major source of support which could continue even after the premature baby is discharged from the ICU.

‘Again the young nurses were quite good at explaining so when this happen we will do x, y and z or this could happen. They were very good I think …’ (CE)

Parents reported conflicting experiences especially with the neonatologists who had treated their premature babies. Some parents expressed frustration, anger and helplessness because they perceived their babies’ neonatologist to be unhelpful. They had less contact with the doctors than with the nurses and when they saw the doctors at their premature babies’ beds they often associated them with trouble. They associated their premature babies’ pain with the neonatologist and they became angry when it appeared as if the doctor did not really care. They expressed a need to be able to see and talk with their babies’ doctors.

‘Dr D, Dr D I like that doctor. He answered my questions … If I had any doubt I would go and see him and ask him.’ (MW)

‘The doctor was very calm. … It calmed me down; it appeared as if he knew what he was doing. … You go with what the doctor tells you and explains to you because you don’t know technology [*sic*].’ (CB)

‘I was angry at the doctors, I said to the doctor help me, write a letter and he basically said, go I have other patients … I was furious.’ (CvS)

The longer the parents spend time in the ICU with their premature babies the more they become aware of the other premature babies, their circumstances and also got to know their parents. They shared experiences, encouraged and supported each other. Parents explained how important it was for them to be seen as competent and coping by the other parents of premature babies in the ICU, but this made it difficult for them to freely discuss their feelings with other parents.

Parents of premature babies in the ICU expressed their need for care, concern, help and support from their family and friends. Some parents felt supported and cared for by their friends and family.

‘My family has been incredible, even though they are all long distance; they have all been on the phone.’ (CE)

However, not all parents were necessarily able to ask for help or discuss their needs with family and friends. This was in some cases related to pre-existing family dynamics. Other parents felt isolated and alone while their premature babies were in the ICU because they had difficulty explaining the different world of the ICU that they and their premature babies spent so much time in. They were wary of their families’ and friends’ reactions to whatever was happening to their premature babies because these often led to more stress and worries for the parents. They were therefore cautious what they would report when they updated their families and friends on the premature baby’s condition. Parents said that they had chosen to focus on the support that they were getting, and on the family and friends who were able to help rather than those who were negative or unavailable.

‘But they have no idea that I was living these two lives and I have no time in between. … Explaining every single step to them over and over again and that also happened with family members and sometimes, some people will say what’s the big deal, your baby is in hospital, being taken care of by the nurses, so what’s the big deal.’ (CE)

‘Then I decided, you know I am not … I have to be strong for H, I had to keep myself positive, I cannot give up I could not allow them to pull me down.’ (CB)

Parents’ spirituality was challenged by their experiences of having a premature baby in an ICU. Parents of premature babies in the ICU tried to find meaning in the experiences they had. They said that not only did their experiences touch their own lives, but it also had a wider impact.

Parents expressed how their relationships with God, as part of their spiritual beliefs, had helped them to have hope and courage for their premature babies. For some parents their spiritual awareness had become more intensified.

‘I spoke all the time with God that He will not take our children. This terrible thing just can’t happen to us.’ (CvS)

‘When I saw the identical twin baby that survived I immediately called her Grace because it was only by the grace of God that she was there. To this day I don’t understand how she could have survived that.’ (CvS)

Some parents found meaning in their experiences because their experiences had given them time to prepare themselves for their baby. Their experiences helped them to challenge old beliefs, re-think their priorities and alter their relationships with reality. It would appear that parents experienced personal growth because of their spiritual experience, but their spirituality was also strengthened because of their experiences.

‘It was actually a very positive experience because I don’t think as a parent I was ready at all, because I knew that ICU is beautiful for us because we get to see the baby but we don’t have to take on all those responsibility all at once. So it was nice having a buffer like that.’ (GE)

## Discussion

The results showed that the premature birth of a baby and the baby’s consequent care in an ICU evoke powerful emotions and thoughts in their parents. The parents’ experiences had a long-lasting effect on them and every relationship that they are involved in.

Lasiuk, Comeau and Newburn-Cook (2013:4–6) confirmed that parents are often unprepared for how helpless their premature babies look and how helpless they themselves will feel in the intensive care environment (Wright 2011:209). They mentioned the fear and insecurity that parents feel. Their fear is often related to whether or not their premature babies will live, and if they live the parents are not sure that they are competent enough as parents (Urden, Stacy & Lough 2006:203). Not only do they have expectations of themselves, but they sometimes have unrealistic and unmet expectations of their babies (Bennett & Sheridan 2005:171), themselves (Leonard & Myers 2008:22; Lewis 2011:73), their partners (Phillips & Tooley 2005:433), the nursing staff (Merighi et al. 2011:1400) and the medical staff (Lasiuk et al. 2013:7).

Strong stress responses were reported by Mendelsohn (2005:197), Steinberg (2006:137), Turan, Bazbakkal and Ozbek (2010:2858) and Elklitt, Hartvig and Christiansen (2007:238). Meijssen et al. (2010:195) explained that parental stress could also be ascribed to a mismatch between the perceived demands of parenting and the resources available.

Some parents were traumatised by their experiences and that left them in a highly aroused state where they were highly sensitive to any changes pertaining to their premature babies. Anxiety and anger are two emotions which parents, as a consequence, experience (Deeney et al. 2009:45; Holditch-Davis et al. 2003:1620; Trombini et al. 2008:894). They often anticipate that their premature babies will regress or even die (Mendelsohn 2005:199; Wakeley, Rae & Cooper 2010:1475). Some parents may want to avoid becoming too attached to their babies (Wright 2011:208).

Relationships play an important role in the experiences of parents with premature babies in the ICU (Cantle 2013:258; Steinberg 2006:137). These relationships include their relationship with themselves, their premature babies, spouses, the medical and nursing staff, friends, family and their spiritual relationships. However, authors (Lasiuk et al. 2013:9; Watson 2011:1469) confirmed that parents have difficulty expressing themselves or voicing their needs. This could be because:

They have guilt-feelings about the premature birth of their babies (Bernard et al. 2011:230; Coppola et al. 2013:1737–1741).

The parents feel that the nursing staff are more knowledgeable than they themselves (Aagaard & Hall 2008:26–36).

They do not get to see their premature babies’ neonatologist to discuss their concerns (Elklit et al. 207:243).

They spend a lot of time at the ICU and they are consequently not able to spend enough time with their support groups, like friends and family to be able to talk about their needs (Greisen et al. 2009:174–1750).

Relationships play an important role in the ‘hope’ that parents have for their premature babies (Shields, Pratt & Hunter 2006:1317–1323). Crain and Koehn (2012:183) supported this but emphasised the importance of the parent’s relationship with the self and the importance of becoming self-aware. Parents have to be self-aware to maintain the hope they have for their premature babies. Parents are also aware which of their other relationships help them to remain hopeful. Weingarten (2010:8, 9) indicated that although relationships are important not all relationships that the parents have will contribute to their hope for their premature babies. Crain and Koehn (2012:182) are of the opinion that the quality of relationships that parents have will be important. The parents’ relationship with nursing staff is particularly important because they are able to educate parents about their premature babies and their needs, they encourage parents and they provide support (Cervantes, Feeley & Larivieri 2011:54–61; Merighi et al. 2011:1400).

Parents’ spiritual relationships are closely related to the hope they have for their premature babies, their future, the meaning that they attach to their experiences and their mental health. Linley and Joseph (2011:151) and Yanez et al. (2009:730) confirmed that when parents find meaning in their experiences, they are more likely to experience mental health.

Parents have various experiences and challenges involving how they feel, what they think and their relationships; some of them are traumatic and some of them are encouraging. However, Barr (2011:127) noted that post-traumatic growth is more likely when parents are able to take responsibility for what they feel.

## Limitations

This research study was conducted in a private hospital where the circumstances and parents’ experiences of their premature babies in an ICU may be very different to that in a public hospital. Participants were either Afrikaans or English-speaking and therefore no African language speakers were included in this research study. Only married people took part in this study; therefore, no single parents’ experiences were explored.

## Recommendations

Recommendations will now be made for nursing practice, nursing education and nursing research. Mindfulness should be facilitated in nurses working in ICUs so that they can promote the mental health of parents with premature babies in ICUs through constructive communication. The promotion of the mental health of parents should focus on parents becoming self-aware and gaining self-knowledge and in the process their self-growth is facilitated.

Nurse educators need to consider facilitating the mindfulness of all nurses during training. When nurses’ practice to become mindful, their own mental health is promoted. Mindful nurses will also be able to render high-quality nursing care.

More research should be conducted in the context of nursing, trauma and mindfulness.

## Conclusion

The premature birth of babies is becoming increasingly common due to various factors and more babies survive their premature birth. Parents of premature babies in an ICU have experiences involving their emotions, thoughts and relationships. These experiences are far-reaching but provide an opportunity for self-growth. They can be used to facilitate the mental health of the parents of premature babies in the ICU. This will also have an impact on the mental health of the premature baby, the family of the parents and society at large. It is therefore in the best interest of parents, their premature babies and society that the experiences of parents with premature babies in the ICU will be utilised to facilitate their mental health.
